# A systematic review of neuroprotective strategies after cardiac arrest: from bench to bedside (Part I – Protection via specific pathways)

**DOI:** 10.1186/2045-9912-4-9

**Published:** 2014-05-01

**Authors:** Dustin B Mangus, Lei Huang, Patricia M Applegate, Jason W Gatling, John Zhang, Richard L Applegate

**Affiliations:** 1 Department of Anesthesiology, Loma Linda University School of Medicine, Loma Linda University Medical Center, Room 2532, 11234 Anderson Street, Loma Linda, CA 92354, USA; 2 Department of Basic Sciences, Division of Physiology, Loma Linda University School of Medicine, 11041 Campus Street, Loma Linda, CA, USA; 3 Department of Cardiology, Loma Linda University School of Medicine, 11201 Benton St, Loma Linda, CA 92354, USA; 4 Department of Neurosurgery, Loma Linda University School of Medicine, 11041 Campus Street, Loma Linda, CA 92354, USA

**Keywords:** Cardiac arrest, Global brain ischemia, Neuronal death, Neuroprotection, Resuscitation, Argon, Xenon, Nitric oxide

## Abstract

Neurocognitive deficits are a major source of morbidity in survivors of cardiac arrest. Treatment options that could be implemented either during cardiopulmonary resuscitation or after return of spontaneous circulation to improve these neurological deficits are limited. We conducted a literature review of treatment protocols designed to evaluate neurologic outcome and survival following cardiac arrest with associated global cerebral ischemia. The search was limited to investigational therapies that were utilized to treat global cerebral ischemia associated with cardiac arrest. In this review we discuss potential mechanisms of neurologic protection following cardiac arrest including actions of several medical gases such as xenon, argon, and nitric oxide. The 3 included mechanisms are: 1. Modulation of neuronal cell death; 2. Alteration of oxygen free radicals; and 3. Improving cerebral hemodynamics. Only a few approaches have been evaluated in limited fashion in cardiac arrest patients and results show inconclusive neuroprotective effects. Future research focusing on combined neuroprotective strategies that target multiple pathways are compelling in the setting of global brain ischemia resulting from cardiac arrest.

## Introduction

The leading cause of death after successful cardiopulmonary resuscitation (CPR) following cardiac arrest (CA) is neurologic injury [1]. In spite of the long-term efforts by the American Heart Association and related organizations to update and disseminate resuscitation guidelines, in-hospital mortality among patients successfully resuscitated remains near 70% [2,3]. For those patients who do survive to hospital discharge, neurologic injury accounts for a significant morbidity with nearly 2/3 of patients having moderate to severe cognitive deficits three months after CA [4].

The aim of this article is to review strategies that could potentially be utilized during or after resuscitation to improve survival and neurologic outcome in patients who suffer CA. Therapeutic hypothermia (TH) is currently recommended by the American Heart Association for comatose patients with restoration of spontaneous circulation (ROSC) after out of hospital cardiac arrest secondary to ventricular fibrillation/shockable ventricular tachycardia (Class 1) and is also considered as treatment for similar patients who suffer in hospital CA and out of hospital CA caused by non-shockable rhythms [5]. The beneficial effects of TH have been published in extensive literature and reviews [6-8] and are not included here. We seek to evaluate other strategies that, when used individually or in conjunction with TH, may further improve neurologic outcomes in patients after CA. The interventions employed during or after CPR that have demonstrated benefits in an animal model of CA may have translation potential to CA patients and thus perhaps provide significant survival and neurologic outcome benefits.

### Literature search method

A literature search was conducted of articles indexed in Medline and published between 1980 and October 2013 using combinations of keywords including “brain injury”, “cardiac arrest”, “neuroprotection”, “cerebral protection”, “cardiopulmonary resuscitation”, “global ischemia”, “global cerebral ischemia”, and “global brain ischemia” (Table 1). Bibliographies of relevant articles were cross-referenced for pertinent articles. Articles were selected for review if postulated mechanisms of neuroprotection and some measure of neurologic outcomes were included. Only neuroprotective strategies tested in animal models relevant to global brain ischemia associated with CA were reviewed. Case reports, pediatric studies and articles not written in English were excluded. Studies of therapies administered before CA were not included as our goal was to investigate potential therapies to improve neurologic outcome that can be employed in the clinical setting (during or after CPR). Similarly, the many studies related to neuroprotection from anesthetic agents were not included, as administration of anesthetic agents during or immediately after CPR may be impractical or associated with undesirable hemodynamic effects. Due to the pathophysiological differences between focal and global cerebral ischemia, the extensive literature regarding neuroprotective strategies in focal cerebral ischemia is acknowledged but not included in this review. In light of the amount of literature available, we have divided this review into 2 parts. Part I of this review (41 articles; Table 2) focuses on approaches that target an individual stage of the cerebral pathological cascade after resuscitation from CA.

**Table 1:** Search terms used to perform literature search

**Database**	**Search terms**
PubMed	brain injury
	cardiac arrest
	neuroprotection
	cerebral protection
	cardiopulmonary resuscitation
	global ischemia
	global cerebral ischemia
	global brain ischemia
	These terms were searched in combinations as subject headings and keywords simultaneously.
	Articles were limited to those printed or translated into English

**Table 2:** Summary of neuroprotective strategies for global cerebral ischemia associated with cardiac arrest

**Therapy**	**Proposed mechanism**	**Study subject**	**Blind**	**Placebo control**	**Rando-mized**	**Delivery route**	**Effect (positive, negative, neutral)**	**Outcomes evaluated**
*Category of Mechanisms I: Modulating neuronal cell death*								
MK-801^15^	NMDA antagonist	Dogs	Yes	Yes	Yes	Intravenous	Negative	Survival^16^, neurological function^15,16^; neurohistopathology^15,16^
GPI 3000^16^								
Lamotrigine^21^	Inhibition of glutamate release	Rats	Not mentioned	Yes	Yes	Intravenous	Positive	Neurohistopathology
Xenon^26, 30--32^	NMDA antagonist	Pigs^26, 30–32^	Yes^26, 30–32^	Yes^26, 30–32^	Yes^26, 30–32^	Inhale^26, 30–32^	Early intervention (10 minutes post-ROSC) Neutral^26^	Neurologic function^26, 30–32^; neurohistopathology^26,^^30,31^
		Human (2 ongoing clinical trials: NCT00879892, NCT01262729)					Late intervention (1 h post-ROSC) Positive^30–32^	
Argon^33,34^	Anti-apoptosis	Rats	Yes	Yes	Yes	Inhale	Positive	Neurologic function; neurohistopathology;
Ischemic post-conditioning ^42,43^	Anti-apoptosis	Pigs	Yes	Yes	Yes	Intravenous	Positive	Survival^43^; neurological function^42,43^; neurohistopathology^43^; Left ventricular ejection function^42,43^
Caspase 3 inhibitor zDEVD-FMK^45^	Anti-apoptosis	Rats	Yes	Yes	Yes	Intracerebro-ventricular	Neutral	Neurologic function; neurohistopathology
Sodium bicarbonate^48,50–52^	Buffering of metabolic acidosis	Dogs^48^	Yes^48,52^	Yes^48^	Yes^48,52^	Intravenous	Positive for long cardiac arrest (15 minutes) and neurtral for short cardiac arrest (5 minutes)^48,52^	Return of spontaneous circulation^48, 50–52^; survival^48,50–52^; neurological function^48,50–52^
		Humans (retrospective^50,51^; perspective^52^; ongoing clinical trial: NCT01377337)	No^50,51^	No^50–52^	No^50,51^			
							Positive at low dose (1 mEq/kg) and negative at high dose (>1 mEq/kg)^50^	Mean arterial pressure and coronary perfusion pressure^48^
							Positive at high usage (dose not specified)^51^	Neurohistopathology^48^
Carbicarb^49^	Buffering of metabolic acidosis	Rats	Yes	Yes	Yes	Intravenous	Positive at low dose (3 ml/kg); Negative at high dose (6 ml/kg)	Mean arterial pressure; survival; neurological function; neurohistopathology
Fluoxetine^55^	Anti-inflammatory	Mice	Yes	Yes	Yes	Intravenous	Neutral at low dose (10 mg/kg); Positive at high dose (5 mg/kg)	Neurologic function; neurohistopathology
Matrix metalloproteinase-9 inhibitor^56^	Anti-inflammatory	Rats	Not mentioned	Yes	Yes	Intraperiton-eal	Positive	Brain water content; neurohistopathology
*Category of Mechanisms II: Influencing oxygen free-radicals*								
Hyperoixa (100%) ventilation^57--62^	Increased oxidative stress	Dogs^57–60^	Not men-tioned^57, 59–62^	No	Yes	Inhale	Negative^57–61^	Neurological function^57–61^; neurohistopathology^58–61^; plasma biomarkers of neuronal damage^62^
		Pigs^61^	Yes^58^				Neutral when co-treated with hypothermia and Negative when not co-treated with hypothermia ^62^	
		Human^62^						
Methylene blue^65–67^	Attenuation of oxidative and inflammatory injury	Pigs	Not mentioned	Yes	Yes	Intravenous	positive	Survival^65^; inflammatory markers^65^; neurohistopathology ^66^; genomics^67^
Inhaled nitric oxide^68,69^	Inhibition of reactive oxygen species	Mice	Not mentioned	Yes	yes	Genotype^68^	positive	Survival^68,69^; neurological function^68,69^; neurohistopathology^68,69^; LVEF^68,69^; brain edema^69^; diffusion weighted imaing^69^
						Inhale^69^		
Nitrite^70,71^	Reversible inhibition of mitochondrial complex I with reduced free radical production^70^	Rats^70^	Yes	Yes	Yes	Intravenous	Positive	Survival; neurological function; neurohistopathology
	Improved mitochondrial function and S-nitrosylation for pro-survival^71^							
		Mice^71^						
N-acetylcysteine^75^	Free-radical scavenger	dogs	Yes	Yes	Yes	Intravenous	Neutral	Neurologic function
*Category of Mechanisms III: Improving cerebral hemodynamics*								
Intrathoracic pressure during CPR^76–79^	Improved organ perfusion	Pigs^76,77^	Not men-tioned^76,77^	Yes	Yes	Intrathoracic pressure regulator^76^	Positive^76–79^	Survival^76–79^; neurological function^76–79^; brain and heart blood flow^76^
		Humans^78,79^	No^78^			Active compression-decompression device + impedance threshold device^77–79^	Neutral for neurologic recovery^78^	
			Yes^79^					
Sodium nitroprusside + active compression/decompression + impedance threshold device^80–82^	Improved organ perfusion	Pigs	Yes	Yes	Yes	Intravenous	Positive	Survival and neurological function^80^; return of spontaneous circulation and carotid blood flow^81,82^; cerebral perfusion pressure and coronary perfusion pressure^81^
Hypertonic saline hydroxyethyl starch^83^	Improve perfusion, decrease intracranial pressure, decrease brain edema	Rats	Yes	Yes	Yes	Intravenous	Positive for cerebral blood flow during early reperfuion; neutral at late time point (7-day post-resuscitation)	Survival; cerebral blood flow; neurological function; neurohistopathology

Superscript numbers indicate the citation number of studies reviewed.

## Review

**Figure 1:**
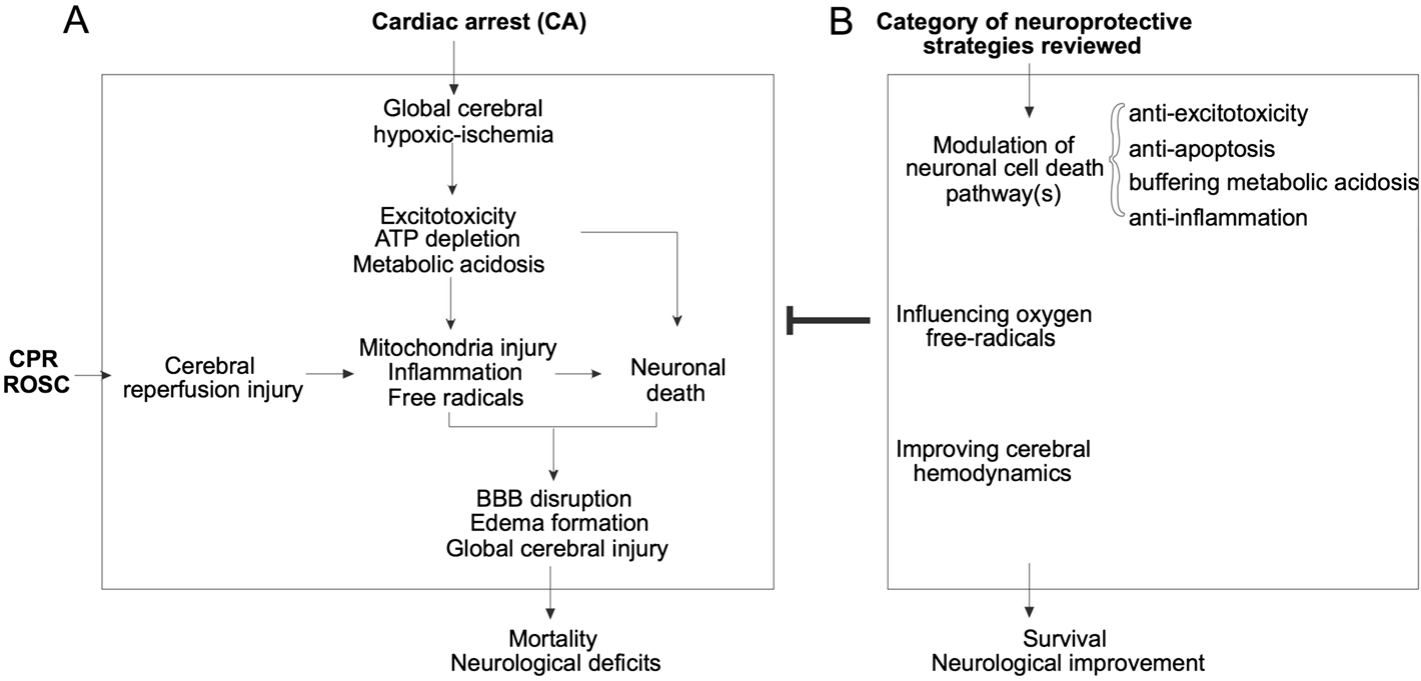
**Pathophysiology and possible mechanisms of protection following cardiac arrest. A**: Comprehensive cascades of pathophysiology constituting global brain hypoxic ischemia and reperfusion injury following cardiac arrest (CA) and return of spontaneous circulation (ROSC). **B**: The possible mechanisms of protection investigated in literature included in this review were separated into three broad mechanistic categories. The effects of these interventions could decrease global brain injury after resuscitation from cardiac arrest and thus potentially improve survival and neurologic outcome.

In CA and resuscitation, the entire brain is subjected to a transient period of complete ischemia followed by reperfusion. The comprehensive cascades of pathophysiology constituting global brain hypoxic ischemia/reperfusion injury are summarized in Figure 1A. Based on the possible mechanisms of protection, the literature was separated into 3 broad mechanistic categories affecting global brain injury shown in Figure 1B: **MODULATING NEURONAL CELL DEATH PATHWAY(S), INFLUENCING OXYGEN FREE RADICALS, OR IMPROVING CEREBRAL FLUID DYNAMICS**.

### Modulation of neuronal cell death pathway(s)

A number of processes can ultimately lead to neuronal injury and cell death following global brain ischemia including excitatory amino acid toxicity, metabolic acidosis and dysregulation of intracellular calcium homeostasis, protease activation, inflammation and activation of programmed cell death pathways [9,10]. Strategies that interrupt the propagation of these cascades would theoretically favor neuronal survival.

#### ***Anti-excitatory amino acid toxicity***

##### 

**N-Methyl-D-aspartate (NMDA) receptor antagonist** Excessive activation of NMDA receptors under conditions of energy substrate depletion results in glutamate excitotoxicity [11]. This pathology has been demonstrated in animal models of global brain ischemia [12,13] and in human patients recovering from CA [14]. Consequently, much effort has gone into targeting glutamate receptor subtypes in an attempt to limit ischemic brain damage.

However, in the setting of global brain ischemia, pre or post CA infusion with the NMDA receptor antagonist MK-801 exacerbated post-resuscitation neurological deficits in the dog model [15]. Similarly post-CA intravenous treatment with the NMDA receptor antagonist GPI 3000 was associated with poor survival and neurologic function along with increased neuronal death in the neocortex and hippocampus in dogs subjected to CA and CPR [16]. An interaction between ischemia and competitive NMDA receptor antagonism may be the cause of deleterious outcomes [15]. Indeed, pharmacological attempts to use NMDA receptor blockers for stroke have had very limited clinical success because these compounds produce additional adverse side effects such as profound psychotomimetic behavioral changes as well as intrinsic neurotoxicity at proposed neuroprotective concentrations [17-19].

##### 

**Lamotrigine** An alternative approach to ameliorate glutamate excitotoxicity can be to inhibit the presynaptic release of glutamate by using the phenyltriazine seizure drug lamotrigine. Lamotrigine has been previously shown to improve survival and neurologic function as well as hippocampal neurohistopathology up to 21 days in a gerbil model of bilateral cerebral artery occlusion [20]. Similar neuroprotection was consistently found in a rat model of CA [21]. Lamotrigine administered 15 minutes after 8.5 minutes of CA significantly improved the number of viable cells compared to non-treated CA rats at 3 weeks after CA. The mechanism of action of lamotrigine is purported to be inhibition of pre-synaptic glutamate release by blockade of voltage dependent sodium channels [22]. Additionally, decreased sodium influx can prevent intracellular calcium overload, further favoring outcome [23].

##### 

**Xenon** The inert gas xenon has both anesthetic properties and a pharmaceutical profile of low-affinity use-dependent NMDA receptor antagonism at nonanesthetic concentrations [24]. It may avoid or reduce adverse side effects and potential neurotoxicity associated with prototypical NMDA receptor antagonists [19,25]. A number of pre-clinical studies have shown potential utility for xenon as a neuroprotectant when administered alone or in combination with TH [26-29]. In a pig model of CA and CPR, combined mild hypothermia (33 degrees C for 16 hours) with xenon (70% xenon and 30% O2 for 1 hour) showed significantly improved neurologic deficit scores over 5 days post-ROSC relative to untreated controls [30]. While neuronal viability was similar to mild TH, combined mild TH + xenon treatment was associated with less astrogliosis and microgliosis, suggesting synergistic protection from the combination.

Other xenon studies have been conducted without combined TH. Pigs underwent 8 minutes of ventricular fibrillation (VF) arrest followed by 5 minutes of CPR [31]; one hour after ROSC the pigs were randomized to 70% xenon in 30% O_2_ for 1 hour or 70% xenon in 30% O_2_ for 5 hours or 70% nitrogen in 30% O_2_ (control). Improvement in neurologic function was transient as benefit was only seen in the first 3 post-ischemia days with no significant difference observed on post-ischemia day 4. At necropsy on day 5, there were significantly reduced numbers of necrotic neurons in pigs ventilated with xenon [31].

A similar study evaluated pigs receiving 70% xenon/30% O_2_ vs. 69% N_2_/1% isoflurane/30% O_2_ vs. 70% N_2_/30% O_2_ for one hour starting 10 minutes after ROSC; findings revealed no differences with respect to neurologic deficit scores over 4 days or neurohistopathological analysis at day 5 [26].

However, a separate xenon study without TH revealed that the time to initiate xenon treatment appears to be critical [32]. Late rather than early treatment with xenon after CA resulted in neurologic benefit. Xenon 70%/O_2_ 30% administered for one or five hours starting one hour after ROSC was associated with improved neurologic function compared to the control group receiving 70% N_2_/30% O_2_[32].

As of this writing, a search of the ClinicalTrials.gov site for xenon and cardiac arrest reveals that there are two human clinical trials recruiting subjects (NCT00879892; NCT01262729). These two clinical trials may provide a better picture about the translation potential of xenon use following CA.

#### ***Anti-apoptosis***

##### 

**Argon** Argon is a non-anesthetic gas that is relatively cost-effective and available compared to xenon. Intriguing data have shown neuroprotective properties of argon in animal models of global brain ischemia secondary to CA. Rats underwent 7 minutes of CA followed by 3 minutes of CPR and one hour after CPR were then randomized to ventilation with 70% argon/30% O_2_ vs. 70% N_2_/30% O_2_ for one hour [33]. Argon treated rats had significantly improved neurologic deficit scores for 7 days and demonstrated statistically better neurohistopathological outcome in the neocortex and the CA3/4 region of the hippocampus. In a separate pig model of CA (8 minutes) and CPR (5 minutes), four hour ventilation with 70% argon/30% O_2_ after ROSC resulted in significantly improved neurologic recovery and less histopathology in brain tissue [34].

In general, the mechanisms of neuroprotection by argon are poorly understood. The mechanism of neuroprotection afforded by argon may involve activation of anti-apoptotic signaling through increasing BcL-xL or Bcl-2 levels, thus promoting cell survival [35]. Argon may also affect gamma aminobutyric acid type A receptors, although further data is required to clarify whether this is the mechanism for cytoprotection [27].

##### 

**Ischemic post-conditioning (IPC)** Although resuscitation requires reperfusion of ischemic tissue with oxygenated blood to restore aerobic metabolism and organ function, reperfusion concomitantly activates multiple pathogenic mechanisms and results in what is collectively known as reperfusion injury [23]. At the center of reperfusion injury are mitochondria, playing a critical role as effectors and targets of injury. Mitochondrial calcium overload can worsen cell damage by compromising mitochondrial capability to sustain oxidative phosphorylation [36] and by promoting the release of pro-apoptotic factors [37]. Although interrupting chest compressions for an extended period during CPR may lead to poor outcome [38], IPC can limit reperfusion injury, thus exerting anti-apoptotic effects through mitochondrial protection [23,39-42]. In pigs subjected to 15 minutes of VF arrest, IPC was given during the first 3 minutes of CPR as 4 cycles of 20 seconds of chest compressions followed by a 20 second pause and compared to a control group of CPR without IPC. Both groups received mild TH for 12 hours after CPR. Left ventricular ejection fraction (LVEF) and neurological functional recovery were significantly better in the CPR + IPC group at 2 days compared to regular CPR. The same investigators further compared the effects of standard CPR, CPR + IPC, CPR + cardioprotective vasodilator therapy (CVT), or CPR + IPC + CVT [43]. CVT (IV sodium nitroprusside 2 mg and adenosine 24 mg) was administered during the first minute of CPR. CPR + IPC showed significantly better 48-hour survival, LVEF, neurological assessment and histological scores compared to the other groups.

##### 

**Caspase-3 inhibitor** Caspase-3 activation plays a central role in apoptotic pathways following brain injury [44]. zDEVD-FMK directly interrupts the apoptotic pathway by inhibiting caspase-3 in focal brain ischemia [45]. However, the same benefits were not observed in the setting of global ischemia secondary to CA. In a rat model of CA and CPR, a 7-day intracerebroventricular (ICV) infusion of the caspase-3 inhibitor zDEVD-FMK after ROSC did not improve neurologic deficit or neurohistopathology at 1, 3 and 7 days [46]. The discrepancy may be attributed to either whole body ischemia/reperfusion syndrome and/or a systemic inflammatory response syndrome, which increases the pathological complexity of brain injury in CA. Additionally, the lack of protective effects may result from high zDEVD-FMK clearance inside the ICV system and/or an impaired uptake into the brain parenchyma [46].

##### 

**Buffering metabolic acidosis** CA prompts a shift to anaerobic metabolism leading to rapid development of intense and sustained intracellular acidosis systemically and locally in the brain. This subsequently triggers a battery of pathological processes resulting in cytosolic calcium overload. In the “low flow” or “no flow” state followed by reperfusion, both CO_2_ elimination by ventilation and metabolic correction by HCO_3_ buffering may be necessary to optimize pH recovery [47]. In this context, buffering metabolic acidosis using sodium bicarbonate (NaHCO_3_) and carbicarb have been evaluated as neuroprotective strategies, but the efficacy remains controversial.

After dogs underwent VF arrest for either 5 minutes (short) or 15 minutes (long), NaHCO_3_ was administered as 1 mmol/kg initially with additional doses as necessary to correct base deficit to -5 mEq/l [48]. Dogs treated with NaHCO_3_ demonstrated equivalent rates of ROSC and 24 hour survival with short arrest but significantly improved ROSC and survival in the long arrest group when compared to controls. Acidosis was also significantly less in the prolonged arrest group treated with NaHCO_3_, while coronary and systemic perfusion pressures were significantly better. Neurologic deficit scores were improved in both groups treated with NaHCO_3_, but histopathologic staining was not different.

Carbicarb is an alkalinizing agent given to combat acidosis inherent to CA. While NaHCO_3_ produces increased CO_2_ and a paradoxical cerebral acidosis, carbicarb is a mixture of HCO_3_ and sodium carbonate that does not produce paradoxical cerebral acidosis. In a rat model of CA (8 minutes) and CPR, low dose (3 ml/kg) carbicarb post-ROSC treatment had positive outcomes for 7-day survival, neurologic deficits and hippocampal cell death compared to controls [49]. The protection was secondary to attenuation of brain pH decreases as well as an increase in post-resuscitation mean arterial pressure (MAP). A high dose (6 ml/kg) group completely neutralized pH but had negative outcomes when compared to the control group with increased neuronal cell death, increased neurologic deficit and decreased MAP [49].

In CA patients, retrospective studies showed some benefits of NaHCO_3_[50,51], but a prospective randomized double blind clinical trial did not [52]. In one of the retrospective human studies, low versus high dose NaHCO_3_ administration during resuscitation had no impact on immediate ROSC but low dose NaHCO_3_ favored long-term outcomes including survival and neurologic outcome [51]. The authors found that administering more than 1 mEq/kg NaHCO_3_ during CPR had a negative impact on long-term survival and neurologic outcome. In another retrospective study associated with the Brain Resuscitation Clinical Trial III [50], patients were included in the study if they suffered out of hospital CA and advanced cardiovascular life support (ACLS) was initiated within 30 minutes by emergency medical services. NaHCO_3_ administration was optional in the study and was preferentially more frequently administered at some institutions than others. Patients treated in the “high NaHCO_3_” user sites had significantly better rates of ROSC and neurologic outcomes. One potential confounder in this study is that the patients who received the higher NaHCO_3_ doses also had a significantly shorter time to initiation of ACLS (1.7 minutes). One prospective randomized double-blinded clinical trial in 875 pre-hospital CA patients showed empirical early administration of NaHCO_3_ (1 mEq/kg) had no effect on overall outcomes in brief (<5 minutes) and moderate (5-15 minutes) CA. However, there was a trend toward improved outcome in prolonged (>15 minutes) CA [52].

As of this writing, a search of the ClinicalTrials.gov site for bicarbonate AND cardiac arrest reveals 1 human trial that is not yet recruiting subjects (NCT01377337). This study proposed to investigate the survival at CPR termination impact resulting from IV administration of 1 mEq/kg NaHCO_3_ after the first IV dose of epinephrine and up to 2 additional doses administered at 5 to 10 minute intervals during CPR.

The discouraging results of attempts to apply this approach from bench to bedside may be due to opposing mechanisms of cellular action. High sodium may help by eliminating H + via the antiporter, but intracellular Na + accumulation may lead to decreased Ca2+ elimination via the Na + -Ca2+ exchanger and Ca2+ accumulation [47]. Ca2+ overload is associated with deleterious cellular consequences that may compromise anti-acidosis protective effects. In addition, acidosis depends on the patient’s medical status before arrest, along with the duration and efficacy of CPR. Treatment may be improved by titration guided by venous or arterial pH measurement. Arrest times longer than 15 minutes will most likely not benefit from bicarbonate administration [47]. Therefore, NaHCO_3_ was recommended for use during CA resuscitation in 1974 but was removed from the CPR algorithm in 1986 secondary to the potential to increase acidosis. The 1992 and 2000 AHA guidelines de-emphasized the use of NaHCO_3_.

#### ***Anti-Inflammation***

Neuroprotective strategies targeting inflammation have been investigated extensively in ischemic stroke [53]. Considering persistent systemic inflammatory responses after CA, anti-inflammation treatment may bring systemic and neurologic benefit favoring improvement in overall outcomes. Only a few anti-inflammation agents have been tested in animal models of CA.

##### 

**Fluoxetine** Fluoxetine is a selective serotonin reuptake inhibitor and has been shown to protect neurons through anti-inflammatory effects in focal brain ischemia [54]. In mouse models of CA and CPR, high dose (10 mg/kg) but not low dose (5 mg/kg) fluoxetine was associated with decreased histological damage in the caudate putamen as well as decreased sensorimotor deficits at 3 days when administered 30 minutes after ROSC [55]. No difference in the hippocampus was observed with either dose.

##### 

**Matrix metalloproteinase-9 (MMP-9) inhibitor** MMP-9 activation plays an important role in blood–brain barrier (BBB) disruption, resulting in brain edema and inflammation following brain ischemia [53]. SB-3CT is a specific inhibitor of MMP-9. In a rat model, CA was induced by occlusion of the airway. CPR was started 1 minute after CA onset. Compared to control CA rats, intraperitoneal (IP) injections of SB-3CT at 5 minutes after ROSC was associated with significantly reduced brain tissue expression of MMP-9 protein and messenger ribonucleic acid, brain water content, Evans Blue content and cytokine levels at 3, 9, 24 and 48 hours [56].

### Influencing oxygen free radicals

Oxidative stress is a common final mechanism of injury contributing to brain damage following CA and ROSC. Excessive production of free oxygen radicals associated with ischemia and reperfusion injury causes cellular lipid and protein degradation. Treatments with mechanisms that reduce free radicals in the brain may be neuroprotective.

#### ***Hyperoxic (100%) ventilation***

Hyperoxic (100%) ventilation during CPR and early ROSC is a traditional component of resuscitation and life support strategies in CA patients. Emerging animal studies have challenged the protective role of hyperoxia in the setting of global brain ischemia with accumulating evidence of aggravating oxidative damage. Dogs underwent 9 minutes of CA followed by CPR and were randomized to resuscitation with normoxia (21% FiO_2_), hyperoxia (100% FiO_2_) or hyperoxia with antioxidant pretreatment [57]. The hyperoxia group had significantly worse neurologic outcomes at 12 and 24 hours compared to both other groups. These results were similar to those in dogs that underwent 10 minutes of CA followed by 3 minutes of open chest CPR and defibrillation [58]. Normoxic dogs were given room air during CPR and after ROSC were ventilated for one hour using room air with higher inspired oxygen only as needed to maintain arterial oxygen between 80 and 100 mm Hg. Hyperoxic dogs were given 100% O_2_ during CPR and after ROSC for 1 hour with no FiO_2_ adjustments. At 24 hours, dogs resuscitated with 21% O_2_ showed significantly decreased oxidized lipids in the frontal cortex and significantly better neurologic deficit scores compared to the hyperoxic dogs [58]. A similar dog study showed a hyperoxic group had significant increase in neuronal death and oxidative damage when compared to normoxic resuscitated (FiO_2_ 21% for one hour, then adjusted to maintain PaO2 between 80–120 mmHg) and shams at 2 and 24 hours after ROSC [59].

In another dog study, glucose metabolism impairment, increase in neuronal death and neuroinflammation were consistently associated with 100% FiO2 ventilation during CPR and after ROSC, exacerbating neurologic deficit [60]. In this study, after ROSC, dogs were ventilated with either 100% FiO_2_ or room air for one hour. The hippocampi of hyperoxic dogs had decreased utilization of isotope labeled glucose at 2 hours.

Retrospective analysis of data from controls in two studies evaluating pigs that underwent 8 minutes of CA followed by CPR was performed [61]. After ROSC, ventilation was with 100% FiO_2_ for 10 minutes or 60 minutes. Hyperoxic pigs had a significantly increased number of necrotic neurons and perivascular inflammation at 5 days after ROSC. There was a trend toward improvement in neurological deficit in the normoxic group but this did not reach significance.

There is one prospective, randomized pilot study in out-of-hospital CA patients with ventricular fibrillation as an initial rhythm that demonstrated the safety of ventilation with low inspiratory O_2_ concentration (FiO_2_ 30-40%) during one hour after ROSC [62]. The use of 100% FiO_2_ may worsen neuronal injury during the early post-resuscitation period in patients not treated by TH [62]. Patients with witnessed out-of-hospital CA were randomized to receive 100% FiO_2_ or 30% FiO_2_ for 60 minutes after ROSC. There was no statistically significant difference in biomarkers of neuronal injury (neuron-specific enolase, NSE, and S-100) between groups up to 48 hours after ROSC. In the subgroup of patients not treated with TH after arrest, there was a significant decrease in NSE among the patients treated with 30% FiO_2_ at 24 hours only. No long-term study has been conducted to evaluate differences in neurologic outcomes in these patients.

Translational use of reduced inspired O_2_ for all patients will be restricted due to the complexity of pre-morbid conditions and complications associated with CA. This low FiO_2_ approach is not feasible in situations where poor oxygenation is likely to occur, for example CA associated with pulmonary edema, near drowning, severe aspiration of gastric contents or pulmonary infection [62].

### Free radical scavengers

Free radical scavengers are drugs that can react with free radicals and yield nonreactive products. Mixed success in preclinical studies of various scavengers [63] and early failures in some clinical trials have diminished early enthusiasm [64].

#### ***Methylene blue***

Methylene blue has been investigated as an antioxidant in the setting of CA. Methylene blue delivered with a hypertonic hyperoncotic solution increased 4-hour survival and decreased plasma inflammatory markers in a pig model of 12 minutes extended CA and 8 minutes of resuscitation [65]. The same group further demonstrated that methylene blue infusion during CPR and continued for 50 minutes after ROSC significantly prevented the disruption of the BBB often seen after ischemia and reperfusion [66], suggesting decreased nitric oxide metabolites. The protective mechanism of methylene blue was also evaluated using the genomic response to CA and treatment with methylene blue in the same study design [67]. This concluded that neuroprotective effects of methylene blue were diverse, involving regulation of soluble guanylate cyclase and other responses that inhibit apoptosis and decrease the inflammatory response.

#### ***Inhaled nitric oxide***

Inhaled nitric oxide (iNO) inhibits and scavenges reactive oxygen species. Mice with varied expression of NO synthase (NOS) underwent 9 minutes of potassium-induced CA followed by CPR [68]. At 24 hours post resuscitation, mice deficient in NOS3 or soluble guanylate cyclase alpha 1 had significantly poorer outcomes while NOS3 deficient mice with cardiomyocyte specific overexpression of NOS3 were protected from the neurological and cardiac dysfunction. It appears that iNO works predominantly on soluble guanylate cyclase and that deficiency of the alpha 1 subunit of this complex undermines the protective effects of iNO.

In a similar mouse model of CA, iNO (40 ppm) at one hour after CPR for 23 hours improved neurologic function at 4 days, LVEF, brain edema and 10-day survival compared to room air mice [69]. The iNO treatment also reduced water diffusion abnormality, caspase-3 activation and cytokine induction [69].

#### ***Nitrite***

During hypoxia and ischemia, nitrite is converted to NO. In a mouse model of 12 minutes of asphyxia induced CA and CPR, significant advantages in survival, neurologic outcome and cardiac function were found in mice treated with IV nitrite [70]. There was decreased mitochondrial oxygen consumption and a reversible specific inhibition of respiratory chain complex 1 of the mitochondria, thus tempering oxidative injury. A follow up study utilized an 8-minute CA model [71]. At 7 days post-ROSC, rats treated with nitrite had a significant survival advantage and reduction in the death of CA-1 hippocampal neurons although not associated with significant neurologic function benefit. This protection was associated with improved mitochondrial function after CA and increased S-nitrosylation for pro-survival signaling.

Regarding clinical applications, establishing the safety profile of these approaches will be necessary due to the dual functional role of nitric oxide. By decreasing nitric oxide metabolites, methylene blue is a potent vasoconstrictor and thus may have limited clinical utility in many patients. Nitric oxide or nitrite may induce systemic vasodilation and hypotension that could preclude their use in CA patients with an unstable hemodynamic condition.

As of this writing, a search of the ClinicalTrials.gov site for nitrite and cardiac arrest returns one trial that is recruiting subjects (NCT01189359). This pilot study investigates the survival following CA impact of 2 micromoles/kg nitrite infusion during CPR. Early results show hemodynamic and methemoglobinemia safety at current doses [71].

#### ***N-acetylcysteine (NAC)***

NAC prevents depletion of glutathione in oxidative injury [72], leading to protection against free radical injury in the liver and lung [73,74]. However, NAC failed to show benefits in a clinically relevant large animal model of global brain ischemia secondary to CA [75]. Dogs underwent 10 minutes of VF arrest followed by CPR. NAC (150 mg/kg) treatment upon ROSC did not improve neurologic deficit scores at 23 hours later. Given that there are many triggers of neuronal injury and more than one final common pathway of neuronal cell death, combination therapies may be required to show functional benefit after global cerebral ischemia. Further pre-clinical research efforts using NAC in combination with other neuroprotective agents may still be worth pursuing [75].

### Improving cerebral hemodynamics

#### ***Intrathoracic pressure modulation during CPR***

In order to improve cerebral perfusion, efforts have been made to enhance CPR efficacy. The mechanical approach using application of modified active compression-depression (ACD) CPR assisted by an intrathoracic pressure regulator (ITPR) and an inspiratory impedance threshold device (ITD) benefitted coronary and cerebral perfusion pressures in a series of pre-clinical studies.

Pigs that underwent VF arrest for 8 minutes were randomized to regular CPR at 100 compressions per minutes or CPR with an ITPR, which combines an ITD with a vacuum source to generate controlled 10 mm Hg vacuum pressure in the trachea while allowing positive pressure ventilation [76]. Use of ITPR during CPR improved all hemodynamic parameters including coronary and cerebral perfusion pressure, blood flow and short-term survival (24 hour) without compromising oxygenation and blood gases. ITPR + CPR increased coronary and cerebral perfusion pressures during hypovolemic CA. Intrathoracic pressure regulation during CPR was re-evaluated in pigs that underwent 8 minutes of VF arrest followed by either standard CPR (S-CPR) at 80 compressions per minute or ACD CPR at 80 compressions per minute plus an ITD (ACD CPR + ITD) [77]. The ACD CPR + ITD group showed significantly improved coronary and cerebral perfusion pressures and improved carotid artery blood flow. ACD CPR + ITD also had significant survival and positive neurologic outcome advantage at 24 hours post-ischemia when compared to animals that had S-CPR.

Although the results from two human studies are not conclusive, ACD CPR + ITD may help improve survival to hospital discharge and short-term neurologic outcomes. In a prospective controlled trial in Germany, patients suffering out-of-hospital CA were randomized to S-CPR or ACD CPR + ITD [78]. Patients in the ACD CPR + ITD group had significantly improved 1 hour and 24 hour survival rates. There was no significant difference in the number of patients that survived to hospital discharge or neurologic score at hospital discharge, although there was a trend towards improved neurologic score in the ACD CPR + ITD group. A study evaluated standard chest compressions during CPR versus ACD CPR + ITD in patients suffering out-of-hospital CA [79]. Survival to hospital discharge with favorable neurologic outcome was significantly better in the ACD CPR + ITD group, as was survival to one year. However, neurologic outcome at one year was similar in all survivors.

Co-administration of the vasodilator sodium nitroprusside appears to further enhance cardiovascular and cerebral hemodynamics [80-82], but these effects have not been validated in randomized patient studies. Sodium nitroprusside (SNP)-enhanced CPR (SNPeCPR) involved ACD CPR with ITD, external application of abdominal force and 1 mg of nitroprusside injection. Pigs underwent 6 minutes of VF followed by CPR [81]. Animals were randomized to one of two groups for 15 minutes of CPR: S-CPR or 5 minutes of S-CPR + SNP then 5 minutes of ACD CPR + ITD + SNP then 5 minutes of ACD CPR + ITD + SNP + abdominal binding (AB). The control group had a significantly worse ROSC while ACD CPR + ITD + SNP + AB showed significantly elevated carotid blood flow when compared to S-CPR. The addition of SNP to CPR did not significantly alter cerebral or coronary perfusion pressures, but did improve carotid artery blood flow. Using the same experimental protocol, significantly improved 24-hour survival, neurologic functional recovery and LVEF were also found in SNPeCPR pigs compared to S-CPR alone [80]. The authors suggested the provocative mechanism was release of nitric oxide causing vasodilation and increasing perfusion to the heart and brain. It is not clear how much of the benefit was secondary to SNP and how much to ACD + ITD. The follow-up study from the same group might help answer this question, although not directly addressing neurologic functional recovery [82]. The study used two protocols. The first protocol randomized pigs to S-CPR, ACD CPR + ITD, or SNPeCPR. Following 15 minutes of VF arrest, CPR was begun and defibrillation first attempted at 6 minutes of CPR. SNPeCPR pigs had significantly better rates of ROSC compared to both other groups (12/12 vs. 0/6 and 0/6). In the second protocol, pigs underwent 10 minutes of VF arrest and were then put into pulseless electrical activity followed by S-CPR or SNPeCPR. SNPeCPR pigs had significantly improved ROSC. In both protocols SNPeCPR had enhanced coronary perfusion pressure, carotid blood flow, cerebral perfusion pressure and end-tidal carbon dioxide.

#### ***Hypertonic saline hydroxyethyl starch***

Early intervention using hypertonic saline hydroxyethyl starch showed some improvement in cerebral blood flow but did not ultimately translate to neurologic benefits in a pre-clinical study using a CA model. The proposed mechanism is that hypertonic saline hydroxyethyl starch improves perfusion, decreases intracranial pressure and decreases brain edema. Rats were exposed to asphyxia induced CA and resuscitation then randomized to receive placebo or 7.5% saline/6% hydroxyethyl starch [83]. The treatment group had increased early cerebral blood flow, but no improvements in 7-day survival, neurologic outcome or neuronal cell death were found.

## Conclusions

Neurocognitive deficits remain a significant source of morbidity and mortality in patients who survive cardiac arrest. Establishing therapeutic options that can be implemented during and after CPR to decrease these neurologic functional deficits is warranted. In addition to therapeutic hypothermia, a number of other modalities involving different mechanisms of action have been tested mainly in the pre-clinical setting using short-term outcomes. To summarize some preliminary conclusions: 1. Xenon and argon modulate neuronal cell death pathways, cross the BBB efficiently and have fast onset making them good candidates for neuroprotection in the setting of cardiac arrest but further study is needed to explore the ideal dosage, initiation time and duration of their applications; 2. Ischemic post conditioning reduces reperfusion injury and may be practical for clinical translation; 3. CPR assisted by active compression-decompression or intrathoracic pressure regulation favors cerebral perfusion; 4. 100% hyperoxia ventilation appears to be harmful after resuscitation.

Several approaches have been evaluated in cardiac arrest patients in limited fashion and the effects on neuroprotection are inconclusive. In addition to further clinical trials evaluating therapeutic neuroprotective treatments, the systemic effects of treatment modalities will also need to be considered as the effects of cardiac arrest and CPR are not isolated but rather have diffuse systemic ramifications.

## Abbreviations

AB: Abdominal binding; ACD: Active compression-decompression; ACLS: Advanced cardiovascular life support; BBB: Blood brain barrier; CA: Cardiac arrest; CPR: Cardiopulmonary resuscitation; CVT: Cardioprotective vasodilator therapy; FiO2: Fraction of inspired oxygen; ICV: Intracerebroventricular; iNO: Inhaled nitric oxide; IP: Intraperitoneal; IPC: Ischemic post-conditioning; ITD: Intrathoracic pressure regulator; ITPR: Impedance threshold device; IV: Intravenous; MMP-9: Matrix metalloproteinase-9; NAC: N-acetylcysteine; NMDA: N-methyl-D-aspartate; NOS: Nitric oxide synthase; ROSC: Restoration of spontaneous circulation; S-CPR: Standard CPR; SNP: Sodium nitroprusside; SNPeCPR: Sodium nitroprusside SNP-enhanced CPR; TH: Therapeutic hypothermia; VF: Ventricular fibrillation.

## Competing interests

The authors declare that they have no competing interests.

## Authors’ contributions

LH participated in design, literature search, evaluation of papers for inclusion and writing of this review. DBM participated in literature search, evaluation of papers for inclusion and writing this review. PMA participated in evaluation of papers for inclusion and writing of this review. JWG participated in literature search, evaluation of papers for inclusion and revision of this review. JHZ participated in the design and revision of this review. RLA participated in design, literature search, evaluation of papers for inclusion and writing of this review. All authors read and approved the final manuscript.

## Authors’ information

LH MD, Assistant Professor, Basic Sciences, Division of Physiology and Anesthesiology, Loma Linda University School of Medicine, Loma Linda, CA.

DBM, MD, Fellow in Adult Cardiothoracic Anesthesiology, Department of Anesthesiology, Loma Linda University School of Medicine, Loma Linda, CA.

PMA MD, Associate Professor of Medicine, Cardiology, Loma Linda University School of Medicine, Loma Linda, CA.

JWG MD, Assistant Professor of Anesthesiology, Loma Linda University School of Medicine, Loma Linda, CA.

JHZ MD, PhD, FAHA, Professor of Neurosurgery, Anesthesiology, Basic Science, Division of Physiology, Director of Neuroscience Research, Associate Chair and Physiology Graduate Program Coordinator, Loma Linda University School of Medicine, Loma Linda, CA.

RLA MD, Professor of Anesthesiology, Loma Linda University School of Medicine, Loma Linda, CA.
